# A Discussion in the Mensanamonographs e-group

**DOI:** 10.4103/0973-1229.32165

**Published:** 2007

http://groups.yahoo.com/group/mensanamonographs/

(The mensanamonographs e-group discusses a number of issues related to medicine, mental health, science, religion, philosophy, man and society. One such it discussed recently was the editorial, ‘To cure sometimes, to comfort always, to hurt the least, to harm never’, by Singh A. R., Singh S. A. (2006), in What Medicine Means To Me, MSM, III: 6, IV: 1-4, p8-9.

*A number of members like Roy Sugarman <beegone@gmail.com>, Vance Berger <bergerv@mail.nih.gov>, D. S. Goel <coldsgoel@hotmail.com>, Nicole <nicole@msryche.com>, Madhukar Bandisode <mbandisode@hotmail.com>, K. A. Kumar <drkakumar@rediffmail.com>, Morten Hesse <hesse@medscape.com>, Sadhu <sadhucmvss@yahoo.co.in>, Shakuntala <shakuntala_a_singh@yahoo.co.uk and Ajai <mensanamonographs@yahoo.co.uk> took part in the discussion. - Eds/.*)

## Debate 2006: To cure sometimes, to comfort always, to hurt the least, to harm never

http://www.msmonographs.org/article.asp?issn=0973-1229;year= 2006;volume=4;issue=1;spage=8;epage=9;aulast=Singh

**Roy, Vance, Col. Goel, Nicole, Madhukar, Kumar, Morten, Sadhu, Shakuntala, Ajai**

My registrars in psychiatry often tell me that they leave medicine to come to psychiatry. Their patients, it seems, require that they be comforted and not hurt. There are two aspects to what might constitute ‘hurt’ in that instance. Firstly, the side effects of medication; and secondly, the pain of behavioral change. The expectations of the clients for what modern medicine can do are largely inflated, as we wrestle with and often win against the world's major diseases. However, medical sociologists tell us that the war against early death has been won by sewerage and hygiene advances, as well as access to nutrition and preventative inoculation, not tertiary care. So there may be wisdom in prevention, not in hurtful side effects or the massive burden of responsible, self-generated change.

Othmer and colleagues (actually family; they are all called Othmer in their article in *Psychiatric Clinics of America*, I think in ’98 or ’99) make a poignant point:

They describe how a woman wakes in the morning to find that her ankles are swollen. She heads off to her physician, who looks at the ankles, then tests her blood and heart to find why the ankles have swollen and produced pedal edema. If, the authors posit, she turned by mistake into the psychiatrist's office, she would have been diagnosed and treated for swollen ankle syndrome.

In another interesting study, in the UK, psychological services were made available for anger management, and 119 patients were referred for CBT. Less than 49 made the first appointment and only 8 of the patients finished the course.

So what is the balance in the psychiatric consultation, the behavioral change interview? It appears that the major incentive to be better is determined by the warmth of the relationship, which can be measured in terms of the outcomes of an empathic stance, namely, reflective listening on the part of the clinician. This alone, rather than confronting behaviors, appears to influence the outcome favorably.

I think comfort, if investigated scientifically, turns out to be more than a goal or an aim of medicine - it's an essential part.

**Roy Sugarman**

Mon.; Sept. 11, 2006; 11:49 p.m.

1. I wonder whether one leaves medicine to come to psychiatry, because psychiatry remains an integral part of medicine, which does not of course mean it's not a psychosocial discipline as well. In fact, being both is what's special about it.
2. Side effects do hurt and are probably part of the therapeutic response. What can have effect can also have side effects. What can comfort carries an inherent risk of hurt as well. However, *harm* has a different connotation. It involves engaging *willfully* in activities that can hurt, with the *motive* of personal gain, unmindful of the needs of the ailing or riding roughshod anyway - like when a doctor knows a medicine has great chances of causing side effects and yet prescribes it, so the sickness increases and he can heroically treat it later. Or, he does not update his knowledge about side effects and prescribes it anyway, since it's new and the pharma company has to be pleased for conference and other sponsorships.
  Medicine, for the man who practices medicine, must mean that the practitioner hurts the least and that he harms never.
3. What the man of medicine must further ensure is, find those modalities of comfort which are possible even when cure is not and also work to make cure possible in the future - hence to comfort always and cure at least sometimes.

**Shakuntala**

Wed.; Sept. 13, 2006; 7:40 a.m.

There is little doubt that many psychopharmacological interventions do more harm than good. The benzodiazepines are a well-known example, and we are now becoming increasingly (and uncomfortably) aware of the flip side of the ‘happiness’ pills, the SSRIs.

Less highlighted, however, are the adverse consequences (and high costs) of many psychological interventions, like psychological debriefing and repressed memory therapy. Conventional wisdom has it that like homeopathy or ‘herbal’ medicine, these are, at worst, mere harmless placebos; nothing could be further from the truth. Many psychotherapists and counselors, perhaps proportionately more than psychiatrists, are inflicting enormous harm, at great public/private cost.

The organized psychiatric fraternity has contributed to the problem by medicalizing normal human emotions/situations, like grief and bereavement; and inventing spurious diagnoses, like PTSD.

I have little doubt that sooner rather than later, we shall prescribe ‘success’/‘happiness’ counseling on the lines of grief (‘unresolved grief issues’) and bereavement counseling.

**D. S. Goel**

Tues.; Sept. 12, 2006; 12:43 a.m.

(*In response to what* Roy Sugarman <beegone@gmail.com> *wrote*)

This is a new idea to me, yet I am not all that surprised to hear it, given the track record of the psychopharmacological interventions, as you mention.

What you are claiming occurs/could not occur if the message getting out reflected reality – that is, if good interventions were *found* to be good and bad ones were *found* to be bad. So the problem seems to be one of accurate evaluation of medical interventions.

What can be done? What can WE do to rectify this problem? Start with an intervention, move on to a study, with data collected and analyzed by those with the most vested interest (and conflicting interests) – from data to results (confidence intervals, ‘*P*’ values), from there to conclusions, and from conclusions to policy.

At which stage do most of the distortions occur? What can be done about it?

**Vance Berger**

Tues.; Sept. 12, 2006; 12:52 a.m.

I am new to this group. I would like to share a personal occurrence as it happens to relate to the posting below.

My background is that of someone who has been on the receiving end of some of these ‘therapies’ mentioned below. I was diagnosed with ‘acute panic disorder and agoraphobia’ when I was in my mid-twenties. I was seeing a nurse practitioner at the time for some strange physical symptoms. The first words out of her mouth when she ‘diagnosed’ my case were, ‘Oh, don't worry, we have a pill for that.’ I am not one to pop pills, but the way I was feeling at that time, I was actually relieved there was a ‘cure.’

At the time, I was too naive to ask many questions; however, I have since learned to be a bit more proactive in my medical care. This ‘magic pill’ instead of relieving my symptoms worsened them to quite a substantial degree. I was unable to then leave my house (or even my couch, for that matter) until the pill wore off. At that point, I decided that pills weren't for me! I talked with another doctor and got involved with a program based on cognitive therapy – which worked great for me in the long run.

What I really needed was to learn about what was going on and to realize that there was no short-term ‘cure,’ which I did. I have since been panic free (well, except for flying in an airplane). The things I learned can be applied to other aspects of my life, and those have been enormously helpful in being in tune with my body and mind through the years.

Thanks for welcoming me to the group!

**Nicole**

Tues.; Sept. 12, 2006; 3:04 p.m.

(*In response to what Col. Dr. D. S. Goel<coldsgoel@hotmail.com> wrote*)

Welcome to the group, Nicole. It's nice to see you interacting right away.

I am sorry to know you had an unpleasant experience with pills. Used judiciously and along with psychosocial therapies, I think they are synergistic rather than contradictory. However, ***judiciously*** is the keyword. It involves both the knowledge skills and the mind set of the clinician. If the former is updated, the chances of hurt are less; if the latter is skewed, the chances of harm are multiplied.

I think the mind set of the treating clinician is the most crucial factor in any therapeutic intervention. It will also determine how abreast he keeps in his knowledge skills. This mind set must involve the firm resolve ***to cure sometimes, to comfort always, to hurt the least, to harm never.*** I think that's the ***mantra*** the man of medicine must adopt today; it encapsulates the Hippocratic and other codes in a small neat sentence.

**Shakuntala**

Wed.; Sept. 13, 2006; 7:56 a.m.

(*In response to what* Nicole <nicole@msryche.com> *wrote*)

### In response to Vance

At which stage do most of the distortions occur? Damned if we know. But the effort must be to check and double check at every step and bring all transgressions to light mercilessly.

### In response to Dr. Goel

I wonder if you can encourage people to seek not just relief from distress but also to promote a sense of well-being through the numerous interactions that modes of psychotherapy promise. I think the positive model of health involves not just absence of disease but also a sense of well-being. What is wrong if psychotherapy becomes a means to this well-being, as long as it is evidence based?

**Shakuntala**

Wed.; Sept. 13, 2006; 8:07 a.m.

(In response to what Berger, Vance (NIH/NCI) [E]” <bergerv@mail.nih.gov> and Col. Dr. D. S. Goel<coldsgoel@hotmail.com> wrote)

I would agree with you, except that this checking – be it double, triple or more – is not sufficient. There is an old expression about *practice making man perfect*, but a more cynical view is that practice simply reinforces bad habits.

Likewise, 20 years of experience may, to a cynic, be one year of experience 20 times. I would humbly suggest that despite the checks and balances built into the system, we merely check the same things over and over again, rather than taking a more comprehensive view of what can go wrong and then checking more broadly.

**Vance**

Wed.; Sept. 13, 2006; 8:10 a.m.

What do you suggest, Vance?

**Shakuntala**

Wed; Sept. 20, 2006; 7:46 a.m.

I suggest keeping an open mind. Many critics of studies step up to report what went wrong. Often, they are ignored; they should not be, nor should their claims be afforded undue credibility. Work is required to verify or refute these claims, but I suggest that this work needs to be done – without fail, every time.

**Vance**

Wed.; Sept. 20, 2006; 6:50 p.m.

You say, ***‘Many critics of studies step up to report what went wrong. Often, they are ignored.’***

They are ignored because it hurts to accept what they say and change accordingly. But hurt is integral to change. Those who want the state of affairs to change must plod on, nevertheless. If they give up or get cynical and pessimistic, the battle for change is lost.

**Shakuntala**

Mon.; Sept. 25, 2006; 8:40 a.m.

True, but how much easier would it be if everyone did his or her part?

**Vance**

Mon.; Sept. 25, 2006; 7:15 p.m.

Vance said, ‘… the problem seems to be one of accurate evaluation of medical interventions. What can be done… what can WE do… to rectify this problem? Start with an intervention, move on to a study, with data, collected and analyzed by those with the most vested interest (and conflicted interests), from data to results (confidence intervals, p-values), from here to conclusions, from here to policy. At which stage do most of the distortions occur? What can be done about it?’

He asks two questions:

‘1. At which stage do most of the distortions occur?

2. What can be done about it?’

Any thoughts?

**Shakuntala**

Mon.; Oct. 2, 2006; 8:20 a.m.

(*In response to what* Berger, Vance (NIH/NCI) [E] <bergerv@mail.nih.gov> *wrote*)

In my experience, the problem largely occurs at the stage of compilation and analysis of the data by those with vested interest and conflict of interest.

In one instance, an investigator intervened in the care and continued the study therapy even when it was not needed. The solution for the first problem would be to arrange for an independent organization, preferably the educational institution under whose umbrella the study is being conducted, to perform compilation and the statistical analysis of the data. Such contract could be developed at the time of the institutional approval of the protocols. In the second instance, it would be very difficult to discover if the inclusion criteria of the study are violated by entering falsified data.

**Madhukar Bandisode**

Mon.; Oct. 2, 2006; 9:36 p.m.

What you say is very likely. But isn't it all counterproductive? I mean, you fudge data, get it published, but ultimately the drug will fail the acid test – patient welfare, and replicability. And the researcher concerned will suffer reduced credibility if he has a string of such publications.

While one can make hay while the sun shines – and public memory is short – we cannot forget that research credibility is built over years and is under constant peer supervision. So, memories are not that short. It is in the researcher's own long-term interest not to fall prey to such shenanigans.

**Shakuntala**

Sun.; Oct. 8, 2006; 6:53 p.m.

While most schools of psychotherapy still aim at what remains the basic goal of curative medical science – removal/alleviation of pain, distress, disease (despite the lip service to ‘positive’ health) - attention is now turning towards what may be called the ‘science of well-being.’ Robert Cloninger reports on this emerging field in his paper ‘The science of well-being: an integrated approach to mental health and its disorders’. (World Psychiatry 2006, 5, 2:71-76). This is available on-line on the WPA website.

**D. S. Goel**

Thurs.; Sept. 14, 2006; 7:57 a.m.

I agree with Dr. Goel. Psychotherapy or pharmacotherapy, either of these, can be harmful when not used judiciously. Errors occur even in expert hands. The possible solution, albeit not easy or absolute, seems to be to avoid making the therapy client menu-driven or therapist menu-driven, but to make it interaction-driven, the interaction being as much therapeutic as each one can handle.

**K. A. Kumar**

Trivandrum - 695 004, India

Thurs.; Sept. 14, 2006; 10:27 a.m.

I think it's just a matter of emphasis due to perceived need, not exclusion. Psychotherapy concentrated more on disease-distress then, since that was what people sought for and the pioneers in the branch perceived was needed.

Now, when well-being is becoming an important concept, psychotherapy has to concentrate on it. In fact, it always had that potential, and the founders of all schools of psychotherapy had it as their ultimate goal. I wonder if *any* school of psychotherapy ever excluded well-being from its agenda. It just, maybe, did not emphasize it as much as the humanist school did.

**Shakuntala**

Wed.; Sept. 20, 2006; 7:41 a.m.

(*In response to what* Col. Dr. D. S. Goel <coldsgoel@hotmail.com> *wrote*)

Dear Dr. Shakuntala Singh,

I perfectly agree. The tragedy is, there is no effort to assess or develop the mind set of the physician in most of the formal training setups. I feel the foundation needs to be laid at the undergraduate level. Then they may start seeking its development, and good teachers can help at the postgraduate and higher level and as they work. But then, where are the good teachers? Or at least how many of them are still around?

I know the medical field; it is quite depressing.

**K. A. Kumar**

Thurs.; Sept. 14, 2006; 10:39 a.m.

1. The reason the mind set is not concentrated on is probably because it will raise many uncomfortable questions about the practices the teachers themselves resort to.
2. So the technical aspects of medical practice are taught in detail. The ethical aspect is conveniently left to personal choice – like a corrupt policeman looks the other way when a crime is being perpetrated.
3. If this is a tragedy, it is of gigantic proportions. The backlash is evident in the form of reduced esteem for the medical professional and numerous lawsuits against them. When commerce supercedes care, what else should we expect?
4. A vigorous soul searching by the medical fraternity is urgently required, if for nothing else, for protecting its own esteem and credibility in the patient's/public eye.

**Shakuntala**

Wed.; Sept. 20, 2006; 7:29 a.m.

(*In response to what* Kumar K. A. <drkakumar@rediffmail.com> *wrote*)

In the context of ***hurt*** and ***harm*** as we have discussed it, maybe we may say psychotherapy and pharmacotherapy may be ***hurtful*** if not used judiciously due to ignorance or honest error of omission or commission, and ***harmful*** if not used judiciously due to lack of updating of essential knowledge or willful acts of omission or commission.

Let me give an example. A doctor gives a medicine for a certain ailment. It is so indicated by research and literature. It causes a severe side effect. The doctor had no previous knowledge from the patient's history or available literature that ***this*** patient could have such a severe side effect; or he has carried out a careful cost-benefit analysis and still feels the benefits to the patient warrant the risks involved; and a severe side effect occurs. This amounts to hurt, not harm.

However, the doctor gives such a medication knowing that such a severe side effect is very much possible and a safer option is available; or has not checked/updated his knowledge about such a severe side effect from available literature; or is enamored of newer drugs peddled by the pharma company without carefully perusing the side-effect profile; and prescribes it anyway. He commits harm, not just hurt.

Similarly, costly diagnostic procedures hurt since they dig a hole in the pocket; however, if they are well indicated, as per research evidence, they are unavoidable and legitimate. But when such costly procedures are not really indicated and are carried out anyway, because the hospital/diagnostic centers have to run and cuts/commissions have to be earned, then they harm.

The dividing line between hurt and harm is known to every clinician. The important thing is whether he can avoid crossing over. That is an important, personal decision to take.

The thesis also is, hurt should, but often cannot, be prevented; so we hurt the least. Harm, however, must always be prevented. There can be no compromise on that. So harm, never.

This is the essence of ethical medical practice.

**Ajai**

Fri.; Sept. 15, 2006; 7:58 a.m.

Dr. Ajai,

‘The best doctor is he who prescribes the least medicine.’

In the present situation, people want to get rid of disease quickly and the doctors also want that their patients get relief as soon as possible. With this mind set, the doctors prescribe medicines of different types which are intended to take care of more than one disease. The question of pinpoint diagnosis does not arise.

Again the patients are subjected to unnecessary diagnostic procedures; therapies; and surgeries such as appendicitis, cesarean section, etc. With the arrival of newer drugs through the so-called MRs to the doctors, these doctors simply prescribe these new medicines without any rationale. Behind all these trends, commercial aptitude and motivation to gain are responsible.

Under such circumstances, chances of doctors having concern for ethical medical practice and issues like the possibility of hurting the patients or causing harm are remote. The moral awakening of the medical fraternity at this juncture is badly necessary.

**Sadhu**

Sat.; Sept. 16, 2006; 8:07 a.m.

Sadhu,

You raise an important point. Therapy may become symptom driven, rather than diagnosis driven. It may also be personal economics and pharma driven. To obviate this, we need to do the following:

1. Make our diagnostic processes more precise. The process at present leaves quite a lot to be desired.
2. Help patients to understand that we are attempting not just ***symptomatic relief*** but ***symptom relief based on a firm diagnosis.*** If the patient becomes a collaborator rather than just a consumer of medical care, rational medical care becomes a reality; in fact, only then it becomes so.
3. While the diagnostic process is on, we attempt to make the wait as less hurtful as possible. So, we carry out only ***necessary*** diagnostic procedures with the ***best*** of implements. We also give symptomatic relief so the wait is bearable. So we hurt the least. But we do not give a readymade cocktail of medication which masks the symptoms and vitiates the diagnostic process, because that harms the diagnostic/therapeutic process.
4. And in the whole process of symptom relief, diagnostic procedures and therapy, at no step should harm be ever attempted.

Medicine thus salvages itself. I have no doubts that much that is good and honorable in the branch can be salvaged if we stop being cynical and do not give up the good fight, present circumstances notwithstanding.

**Ajai**

Sept. 16, 2006

Ajai,

While going through the ‘comfort, hurt and harm trio,’ I have developed curiosity about these terms. Actually we should not limit our discussion *just to treating patients* with comfort, hurt or harm as outcome. During history taking or explaining some queries or examining the patients at bedside, we may hurt or harm them. Due to lack of communication skills, many of our physicians hurt the patients and their attendants, and in return they invite trouble from the public. In this regard, let me tell you one event which occurred in 1993. I was working as an M.O. in District HQ Hosp., Bolangir, Orissa. Some patients were admitted after meeting with an accident. One of them had sustained head injury. One attendant asked the surgeon about the prognosis of that patient. The Asst. Surgeon present there said that the patient would go to heaven. The way he said this unpleasant truth irritated the attendants and they were after that doctor. He had to hide for 3 days.

So, while dealing with patients, we may not only hurt or harm them. We may do so to their attendants too. And starting from history taking, examining, laboratory test or diagnosing and daily checkup, we make them comfortable by showing our concern, sweet words, warm touch; or else, we may hurt them emotionally or harm them.

Some materials related to ‘hurt and harm dilemma’ are attached, though I know very well that you as a psychiatrist are aware of the meaning of *hurt* and *harm.* Going through the meaning of these terms, I found a lot of differences and confusion regarding these terms.

**Sadhu**

Sun.; Sept. 17, 2006; 8:04 a.m.

Sadhu,

The definitions are a welcome addition to the discussion, Sadhu. Dictionary definitions sometimes help clarify issues. But they are inadequate to clarify concepts fully.

Let me clarify what we mean by hurt and harm. By hurt we mean the physical, emotional and even social distress caused to a person acted upon - in this case, the patient. Even when a doctor injects a patient, it hurts. However, the doctor's intention is not to harm but to help the patient recover - even if in the process, he adopts procedures that hurt. However, when the intention becomes *mala fide,* it amounts to harm. Harm is the physical, emotional and social hurt which results because the perpetrator does not have the *intention* to relieve distress; rather he *intends* to add to it. When a doctor injects even if it is not indicated or causes exacerbation of symptoms by his actions, either willfully or due to deficient knowledge, he not only hurts the patient but also harms him.

We will immediately realize that hurt is always acceptable to patients. It is only when they get the feeling of being harmed that they resent it. And most actions against the medical profession, whether legal or otherwise, are due to this perceived feeling of harm.

It's when the patients and their advocates feel the *intention* of the medical man was *mala fide* that they rise in protest and want to hurt back.

**Ajai**

Mon.; Sept. 18, 2006; 7:53 a.m.

Having read the article by Berger and Ioannidis in BMJ (http://bmj.com/cgi/content/full/329/7480/1436), I must say that Ajai is being overly optimistic. Honesty may not be the best career move. I personally find myself in a lucky spot, where I can largely say what I want and get a position on less prestigious publications, but I am afraid that there are too many interests at stake to get all the lies and deceit in research out in the open. Editors are interested in covering up bad research once it has been published in their journals, institutions are interested in covering up fraud and co-researchers will be drawn down on their own once fraud and fabrication come out.

**Morten Hesse**

Mon.; Oct. 9, 2006; 2:55 p.m.

Morten and friends,

It's I who made that remark, not Ajai. Though I have strong reasons to believe he would agree.

I share your anguish when you say that honesty may not be the best career move. But I do not share its conclusion. If it means we should stop being honest, I think we accept the fight is over and lost. Often the good intentioned accept defeat too easily. The fight for the right is perennial and unrelenting. Just as they have no choice but to manipulate, we have no option but to remain committed to a firm foundation of values. The fence sitters and turncoats are inconsequential.

Now, I must take leave as I have to go to lead the rally of our institution for the Mental Health Day. Hope the rest of you do your bit too.

**Shakuntala**

Tues.; Oct. 10, 2006; 8:20 a.m.

Just to make sure - I draw the same conclusion as Shakuntala. Have a good Mental Health Day.

**Morten**

Tues.; Oct. 10, 2006; 1:07 p.m.

I couldn't agree more!

**Vance**

Tues.; Oct. 10, 2006; 9:11 p.m.

(*In response to what* Morten Hesse<hesse@medscape.com> *wrote*)

I found Vance's write up delightful. Thanks for bringing it to our attention, Morten. It is one more facet of Vance which most of us didn't know about. However, if it is meant to arouse pessimism about the goings-on in the world of medicine, it will be counterproductive. I think it is more to expose the nefarious goings-on and join the good fight against it.

**Ajai**

Wed.; Oct. 11, 2006; 7:33 a.m.

I'm not sure that I fully comprehend the distinction between pessimism on the one hand and on the other hand recognizing how it is that the public is being raped, on a daily basis, by those involved in medical research. Clearly this is not something that every medical researcher is doing, nor can I claim that it is even a majority (although for all I know - and for all YOU know - it could be). But sitting on the sidelines not doing what you (note: this is a generic ‘you’ and not Ajai) can to combat this problem makes you (generic) part of the problem. Who cares how precisely we can measure something if the effect will only go on to get overwhelmed by biases introduced intentionally or by accident?

Let us do what we can to clean up the infrastructure. I am no physician, but even I know that prior to treating a laceration, one must clean it up. Let us clean up this problem so that we can see what other problems remain to be tackled.

**Vance**

Wed.; Oct. 11, 2006; 7:45 a.m.

Vance,

Pessimism makes one resign to the inevitable. Awareness makes one rise up to join the good fight. I definitely espouse awareness, which makes one want to do something about the nefarious goings-on. I do not accept pessimism, which makes one just smirk sitting on the sidelines. I think you will agree this needs to be done. Hence I would also agree with Shakuntala when she so insightfully said that, to that extent, the fence sitters and turncoats do not matter. This is indeed a perennial and unrelenting fight, and one better decide which side one would like to be counted on.

**Ajai**

Wed.; Oct. 11, 2006; 8:06 a.m.

Yes, I would agree with that!

**Vance**

Wed.; Oct. 11, 2006; 6:39 p.m.

That's what I thought. I share your anguish, but I think you and I and so many like us are ready for the protracted fight for the good. Results may not be forthcoming that easily or soon. That only makes the fight so much more exciting, Vance.

**Ajai**

Thurs.; Oct. 12, 2006; 8:06 a.m.

I'm glad that you are enjoying the trip, but the destination is not the be-all and end-all. On the way, the streets are lined with the bodies of the collateral damage. While we eat, drink and make merry, bad research leads to distorted results, which lead to distorted guidelines, which lead to patients taking treatments that are not ideal for them. The resulting morbidity and mortality is inevitable. Society cannot afford the status quo.

**Vance**

Thurs.; Oct. 12, 2006; 8:18 a.m.

It's a protracted battle, Vance. I did not say I am ***enjoying*** the trip. I said that the results are not so easily forthcoming, which makes the fight more ***exciting***. By *exciting*, I mean it gets one ready to fight further. We do not eat, drink and make merry while the patients are left to the hoodlums of research. We fight the battle, ***each in his own way and to the extent of his capacities***, while not resigning to cynicism or pessimism. Because that is what the hoodlums would want – that we resign to pessimism and cynicism. Then their battle is won. But I do not think while we pursue the right relentlessly, we need not enjoy life as well. Serious pursuits need not be carried out in a glum manner. Also, I do not favor the thought that all research is tainted. There is good work being done, which alone will stand the test of replicability and patient welfare, the only two real pillars of biomedical advance. And I fully agree with you that we cannot afford the status quo. Also, we neither accept distorted results and distorted guidelines, nor do we accept the resultant morbidity and mortality. We work to remedy things, each in his own way, in spite of numerous constraints and roadblocks.

(The Academia-Industry Symposium of the ***MSM*** was an effort in that direction. See http://mensanamonographs.tripod.com/id87.html and the pages that follow there. That was our contribution, a drop in the ocean, to the effort needed to carry on the good fight. And the next *MSM* to follow will detail some of the other areas which need urgent correction. See http://www.msmonographs.org/forthcomingmsm.asp).

But all this can only happen only if we do not become pessimistic and resign to the state as it obtains. Pessimism and resignation – count me out of that. I am sure you would not like to be counted in as well, as would most of us here.

**Ajai**

Thurs.; Oct. 12, 2006; 9:17 a.m.

I'm with you. I just wish there were more among our ranks to fight this good fight.

Vance

Thurs.; Oct. 12, 2006; 9:38 p.m.

Vance,

O yes, there are. I always knew your heart was in the right place, Vance. And I always also liked a stanza of Kipling's poem ‘IF’ (I quote from memory):

*If you can keep your head*
*When all about you are losing theirs*
*And blaming it on you;*
*If you can trust yourself when all men doubt you*
*But make allowance for their doubting too;*
*If you can wait and not be tired of waiting,*
*Or being lied about, don't deal in lies,*
*Or being hated, don't give way to hating*
*And yet don't act too good, nor talk too wise.*

**Ajai**

Fri.; Oct. 13, 2006; 8:11 a.m.

Could you say something more about your thoughts? Here are a few of mine. There are a lot of obstacles, ranging from career fears, general shyness and insecurity for oneself (so how good/unbiased is really my own research) to reluctance to hurt colleagues' feelings. And there's also a bit of fear about doing damage by raising doubts about research in general. You know the kind of argumentation: ‘All these studies were flawed; how about the studies that showed that cigarettes kill people? Could there be something with them too? Maybe, it's just the public stigma that makes people get sick. I'm not going to be misled about that if I like my cigarettes.’ ‘Maybe this herbal cure will deal with the tumor just as well as the operation. Who knows what big pharma went into the studies that said that doctors could cure cancer? I can think for myself and let my biopathic doctor treat me.’

This is not an argument against doing something, but should it be considered? I'm just wondering. And if so, how? Any thoughts on ‘to harm never’?

**Morten**

Fri.; Oct. 13, 2006; 2:20 a.m.

If no study is perfect, why carry out studies at all? Because decisions will be made with or without the studies to inform them. Making decisions without data is still one form of decision making, albeit not a very good one. Imperfect information is generally better than no information. Furthermore, there are shades of gray among imperfect studies. Some are still more reliable than others.

**Vance**

Fri.; Oct. 13, 2006; 2:34 a.m.

Morten raises a very important issue. I request members to respond, as Vance already has.

**Ajai**

Fri.; Oct. 13, 2006; 8:20 a.m.

Hi,

I think, upon reading my mail again, it was not very clear. The question is this: How do we raise the problem of poor research in public without adding to the kind of skepticism that says, ‘if these studies are flawed, maybe all of science is flawed.’?

Greetings,

**Morten**

Fri.; Oct. 13, 2006; 10:37 a.m.

Thank you, Ajai, I love that poem! In fact, maybe I will share one of my poems, recently published, as the subject may be of interest, even though it has nothing to do with medical research:

**Global Warming**

- Vance Berger

And here in the museum you'll find
This thing we call a coat.
Coats are obsolete today,
As surely you will note.
There was a time when it got cold,
Years ago, the days of old.
But now the sun has broken through.
There's nothing left for us to do,
But reminisce about the days,
When ovens still were used for blaze.
No more are there any stoves,
Yet still we cook our garlic cloves.
Simply place them in the street,
And let them soak up all the heat.
I just cooked a stroganoff,
But I can't turn the oven off.
Eternal sauna is too much,
The ground is way to hot to touch.
The polar caps now H2O.
No place left for us to go.
So hurry now and don't be late.
I fear the bus just will not wait.
Make sure all your bags are packed.
No way we are coming back.
We had our chance to keep this place,
But now must fly to outer space.
I'm sorry if I made a fuss,
But now we ride the Pluto Bus.
http://baltimorechronicle.com/2006/041706Berger.shtml

**Vance**

Fri.; Oct. 13, 2006; 6:49 p.m.

(*In response to what* Ajai Singh<mensanamonographs@yahoo.co.uk> *wrote*)

Morten has reframed his question and is waiting for the group to carry the discussion forward. I request you to do so.

**Ajai**

Sat.; Oct. 14, 2006; 11:34 a.m.

One more dimension of Vance comes to the fore. Good effort, Vance.

You and all friends here should know that ***MSM*** has a column called ***MSM Poems,*** where we publish poems related to medicine, health and human behavior. Savor these:

### A case of identity

http://www.msmonographs.org/article.asp?issn=0973-1229;year= 2006;volume=4;issue=1;spage=208;epage=209;aulast=Singh

### More smoke

http://www.msmonographs.org/article.asp?issn=0973-1229;year=2006; volume=4;issue=1;spage=210;epage=210;aulast=Singh

**Ajai**

Sat.; Oct. 14, 2006; 11:32 a.m.

(*In response to what* Berger, Vance (NIH/NCI) [E] <bergerv@mail.nih.gov> *wrote*)

Morten,

Instead of concentrating on, and discussing what, is poor research, it is important we discuss and lay down criteria of what good research is. What does not follow it, is automatically poor research. By keeping this perspective, we will not overbalance or keep concentrating on poor research all the time. Thus the perception in people's minds that all research is poor will not result. Let me put it a little differently. Instead of concentrating on who is a bad doctor, bad teacher, what is a bad marriage, etc., if we concentrate on who is a good doctor, good teacher or what is a good marriage, we will understand the positive, constructive aspects of all these institutions and never allow our perspectives to be skewed. We will also get the best out of these institutions as per the demand of the times. Along with this, we will also know that some percentage of that which is bad is also present, which needs to be understood and exposed. But we will never get pessimistic or cynical and blanket all research or institutions as bad. It is all a matter of perspective and not allowing our minds to be biased and unduly alarmed. Much that is researched is genuinely good. This must always be highlighted. The poor is automatically consigned to oblivion, at least with the passage of some time.

**Shakuntala**

Sun.; Oct. 15, 2006; 8:12 p.m.

That's great. Now can somebody please show me one clinical trial that was designed, conducted and analyzed in an appropriate manner?

**Vance**

Sun.; Oct. 15, 2006; 11:36 p.m.

Sobering thought, Vance.

My response -

It is possible to find fault with any and every clinical trial, because there is human instrumentality involved. That's not the point. The point is, can we know if the fault was intentional or not? What proves that in science?

Two prime methods:

1. Replicability
2. Patient welfare

Any clinical trial of any nature which stands these two tests is a step forward, even if it may be found faulty later. A trial with *mala fide* intentions will be exposed because it will not stand these two crucial tests. Now, we can look into the literature of clinical trials and see which are of this type. Naming one and not the other unwittingly casts aspersions on the latter's legitimacy. So I would not name any. But each one can apply this rule to judge for oneself.

**Shakuntala**

Tues.; Oct. 17, 2006; 7:45 a.m.

It seems to me that only two things emerge from research: variance as a result of main effects and variance from error. Good research is that which has little error variance and well-explained and predicted main effects variance. Bad research is everything else!

**Roy Sugarman**

Tues.; Oct. 17, 2006; 9:18 a.m.

You are correct, but a few points seem relevant to this discussion. First, I am talking only about errors that are easily corrected without introducing additional research costs. So this is not some esoteric discussion of how one can never really know the truth. That is true, of course, but I am limiting my consideration to steps that can be taken to produce more reliable results (as in, more reflective of this unknown truth). And I am not even talking about larger sample sizes or loner studies. Again, I consider only measures that do not introduce additional costs. Even this much seems to be asking too much.

Second, when errors are made, they tend to be made so as to favor the experimental group. That has to give one pause in attributing these errors to honest mistakes. Allowing pharmaceutical firms to run their own trials is similar to allowing builders to then serve as building inspectors and inspect their own buildings. I am not sure that any real improvement will occur until the sponsors fund research indirectly, by putting money into a fund that then goes to some independent third party to actually conduct the testing.

**Vance**

Tues.; Oct. 17, 2006; 8:39 p.m.

(*In response to what* Shakuntala Singh<shakuntala_a_singh@yahoo.co.uk> *wrote*)

In addition to variance, there is also bias.

**Vance**

Tues.; Oct. 17, 2006; 8:44 p.m.

(*In response to what* Roy Sugarman<beegone@gmail.com> *wrote*)

Your comment is too cryptic for comfort. Expound on it, Roy.

**Ajai**

Wed.; Oct. 18, 2006; 7:04 a.m.

Vance,

I know you wish to correct some obvious errors, where no additional costs may be involved, by some simple, easily adoptable measures. Do you also realize why they are not (and will not be) carried out, even when people know they may work? Errors are not that easily corrected, Vance, if we do not have a clear vision about where we are heading.

What I was suggesting was nothing esoteric at all. It was very practical - if only we stop to ruminate a bit, if only every biomedical researcher knew that he will not be judged only by where and how much he is published, but his work will ultimately be judged by:

1. Whether others can replicate it and find it to be true as well
2. Whether it brings about patient welfare

Then he will be forced to engage in worthwhile research. If we as researchers and observers of research also apply this yardstick, we will be able to sift the chaff from the grain. Now, when such is the atmosphere, no researcher adopts dubious means or cuts corners, in methodology or statistics.

Just take an example of research that is still quoted after a few years or which guides future research after it is published. It is only the one that stands these two crucial tests; all the rest are straws in the wind - what we, in India, would call ‘the *Aya Rams* and *Gaya Rams* of research.’ Then neither pharma control nor pliant researchers nor mediocre research will rule. The barometer is there for all to check on. The remedy lies somewhere else, Vance. The malady needs not symptomatic but diagnostic treatment.

**Shakuntala**

Wed.; Oct. 18, 2006; 7:42 a.m.

I think I agree - that sounds reasonable!

**Vance**

Wed.; Oct. 18, 2006; 6:45 p.m.

Thanks for agreeing Vance. Morten's question still needs some more responses from the rest. Just to recapitulate what he asked - *How do we raise the problem of poor research in public without adding to the kind of skepticism that says, ‘if these studies are flawed, maybe all of science is flawed.’?*

**Shakuntala**

Sat.; Oct. 21, 2006; 1:14 p.m.

I wish to pick up the threads of a discussion we had earlier. Morten asked a very valid question when he said -

How do we raise the problem of poor research in public without adding to the kind of skepticism that says, ‘if these studies are flawed, maybe all of science is flawed.’?

We must be critically aware that in trying to uncover scientific faults and misconduct, we do not overbalance and condemn the whole scientific enterprise itself. For this we must remember that when we draw up a balance sheet of scientific developments, we do not forget to add the numerous ***assets*** of science in our obsession with listing its liabilities. This is the duty as much of the critics as the proponents. The critics may be obsessed with finding fault; the proponents must ensure they drive home its good points - and the intelligent observer must never get taken for a ride by ***either.***

We know science is a mixed blessing. We wish to add to its potential for good while subtracting from its equally great potential for evil. In how well we do so rests the future progress of mankind, as of the branch itself.

Counterbalancing forces like religion, philosophy, ethically aware scientific peers and social activism must remain eternally vigilant to the perils of scientific progress. Equally important, the proponents and torchbearers of the glorious tradition that scientific progress represents must ensure that science continues to progress on the bedrock of evidence and social welfare. And while the former must never relent in devising numerous checks and balances, the latter must equally never relent in the pursuit for a better, more evidence-based and beneficial future for mankind that scientific progress has the distinct potential to ensure. That is the challenge and opportunity for both sides, provided they realize this and are able to rise to the challenge. This also, in a way, encapsulates the present predicament of man and italicizes the potential that scientific progress carries for today's society, as also for its future progress, even survival.

I leave you with this thought on the last day of this eventful year, as we all get ready to welcome the new one with traditional gusto, but equal apprehension.

A mixed blessing, again. And why not? Isn't life itself a mixed blessing? As the poet so poignantly says -

***Kahin kisiko mukammal jahan nahin milta…*** (No one ever gets a complete, utopian world…).

Happy 2007, nevertheless!

**Ajai**

Sun.; Dec. 31, 2006; 02:22:47 +0000 (GMT)

